# BioShell 3.0: Library for Processing Structural Biology Data

**DOI:** 10.3390/biom10030461

**Published:** 2020-03-16

**Authors:** Joanna M. Macnar, Natalia A. Szulc, Justyna D. Kryś, Aleksandra E. Badaczewska-Dawid, Dominik Gront

**Affiliations:** 1 Faculty of Chemistry, Biological and Chemical Research Center, University of Warsaw, Pasteura 1, 02-093 Warsaw, Poland; joanna.macnar@student.uw.edu.pl (J.M.M.); natalia.a.szulc@gmail.com (N.A.S.); juchxd@gmail.com (J.D.K.); dawid.aleksandra@gmail.com (A.E.B.-D.); 2 College of Inter-Faculty Individual Studies in Mathematics and Natural Sciences, University of Warsaw, Stefana Banacha 2C, 02-097 Warsaw, Poland; 3 Laboratory of Protein Metabolism, International Institute of Molecular and Cell Biology in Warsaw, 4 Ks. Trojdena Street, 02-109 Warsaw, Poland

**Keywords:** software, structural bioinformatics, macromolecular structure analysis, python library

## Abstract

BioShell is an open-source package for processing biological data, particularly focused on structural applications. The package provides parsers, data structures and algorithms for handling and analyzing macromolecular sequences, structures and sequence profiles. The most frequently used routines are accessible by a set of easy-to-use command line utilities for a Linux environment. The full functionality of the package assumes knowledge of C++ or Python to assemble an application using this software library. Since the last publication that announced the version 2.0, the package has been greatly expanded and rewritten in C++ standard 11 (C++11) to improve its modularity and efficiency. A new testing platform has been implemented to continuously test the correctness and integrity of the package. More than two hundred test programs have been published to provide simple examples that can be used as templates. This makes BioShell an easy to use library that greatly speeds up development of bioinformatics applications and web services without compromising computational efficiency.

## 1. Introduction

Bioinformatics is a field of research inherently related to use of vast data that are produced by biomedical and biological research. The ultimate goal of bioinformatics is to create a system that can help convert a huge amount of data into knowledge. The software inadequacy is currently the major bottleneck that impedes this process. Virtually every novel methodology has been published as a stand-alone program, a web server, or an extension of an already existing package. The software inventory of the field has been extensively growing in the past few decades, but only very few packages are widely used. Numerous specialized tools have been published, as well as general utility software libraries and scripting environments [1], most notably (in the order of the first publication): BioJava [2,3], Biopython [4,5], BioPerl [6], BioShell [7,8], and BioRuby [9].

The first version of BioShell was released in 2006 as a set of command-line utilities. Later, the package was reimplemented as a library for Java programming language [8]. Since then, the package has been extensively used by its developers in daily research tasks. The package has been also used by other research groups, primarily in tasks related to protein structure prediction and modelling. Chowdhury [10] studied the structural ensembles of wild-type systemin plant hormone along with its 17 variants with replica-exchange molecular dynamics. BioShell was used for crmsd calculations and hierarchical clustering of hormone conformations. In two other works, Alvarez et al. explored the applicability of novel methods for protein structure prediction [11,12]. They implemented their methodology as BioShell scripts and concluded that “BioShell combined with the methodology presented in this paper, is crucial in order to predict protein structures while avoiding structural clashes”. In yet another work done by Abagyan [13] group, BioShell was used to reconstruct atoms and larger parts of chemical groups missing in protein structures. In this contribution, we present the newest version, rewritten in C++11, which provides widely extended functionality. While developing BioShell, we conformed to good practices of software development, including continuous integration, unit testing, and code review. Our adherence to these practices makes BioShell suitable for inclusion in major bioinformatics pipelines, database systems, and software projects. Extensive documentation and numerous detailed examples, published on ReadTheDocs website, makes the toolkit easy to approach.

## 2. Methods

The C++11 programming language was chosen to implement the library due to the many handy features the language and its standard library provides, most notably smart pointer implementation. BioShell also relies on multithreading and regular expression support provided by the standard C++11 library. Standard containers (such as std::vector or std::map) are used where possible. The C++11 standard is by now very matured, supported by common compilers, and highly portable.

### 2.1. Command Line Utilities

Since its first release, the BioShell suite has provided a set of command line applications for the analysis and manipulation of protein sequences and structures, such as clust for hierarchical clustering (previously published as HCPM—Hierarchical Clustering of Protein Models) [14,15] and seqc and strc, sequence and structure converters, respectively. These programs are controlled by command line options, which allow users to provide input data and specify the desired output. In the current release, the core applications are supplemented with an over a hundred small utility programs. These utilities also serve for testing purposes, as discussed below, and follow the “one task-one app” paradigm. Each of them performs a particular, very well-defined action. Altogether, the programs included in BioShell distribution were deliberately chosen to solve many daily problems, such as converting a file from one format to another or gathering statistics of structural properties measured on a set of input PDB files. A large collection of examples using these applications are provided in the BioShell cookbook, published on the ReadTheDocs website (https://bioshell.readthedocs.io). In the case of very sophisticated or more custom problems, these programs, however, may not offer a ‘from the shelf’ comprehensive solution and writing a custom program or script calling BioShell library functions may be necessary.

### 2.2. C++ Software Library

**Figure 1:**
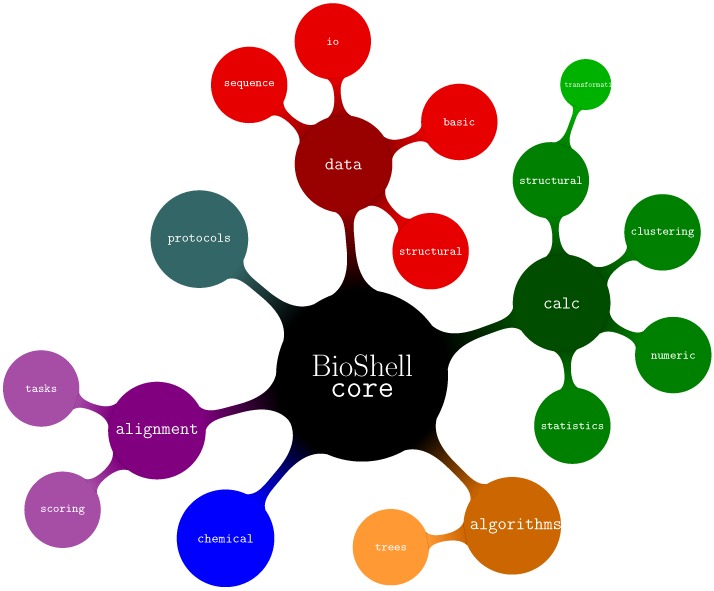
Organization of BioShell source tree—core namespaces.

BioShell source code has been divided into three top-level namespaces: core, ui, and utils, with the first of them being the most important for users as it provides the actual bioinformatics functionality. The submodules of core (see Figure 1) are:

- algorithms—several algorithms used by BioShell such as Union Find, routines to work on trees and graphs.
- alignments—classes related to storing, assessing, and computing alignments between sequences as well as protein structures.
- calc—calculations on biomacromolecular structures (core::calc::structural), data clustering (core::calc::clustering) and generic numerical and statistical routines.
- chemical—classes representing biochemical concepts such as atoms and amino acids
- data—I/O routines core::data::io, data representation of sequences. core::data::sequence and structures core::data::structural, generic data types such as 3D vectors and specialized matrices core::data::basic.
- protocols—classes optimised to perform specific, computationally demanding tasks such as pairwise crmsd calculations. The actual computations are performed by modules from other namespaces (primarily from core::calc::structural). It might be easier for a user to directly employ the latter for small-scale computations. For large scale projects, however, the protocols submodule provides mechanisms for distributing jobs between threads, filtering results and other post- and pre-processing operations.

### 2.3. Python Library

This current release of BioShell package also provides bindings to the Python scripting language. We use a binder tool (https://github.com/RosettaCommons/binder) to automatically generate binding code. The majority of BioShell C++ classes are available as modules in Python, with a few exceptions of C++ templates. Python nested sub-modules correspond to the C++ namespaces with a pybioshell prefix, e.g., the C++ class that is responsible for loading PDB files (core::data::io::Pdb), is accessible from Python as pybioshell.core.data.io.Pdb.

## 3. Results

### 3.1. Improved Performance

Substantial effort has been devoted to optimize the computational efficiency of BioShell routines. In several cases, this has an influence on the software architectural design. Here, we discuss in detail the loading of PDB files as an example. Reading and parsing biomacromolecular structures in the PDB format are fundamental tasks in structural bioinformatics. In fact, parsing these files often takes more time than subsequent calculations and may become a bottleneck when a very large number of PDB models is required for analysis.

In order to store biomacromolecular data, the BioShell library implements a hierarchy of classes that is similar to what can be found in other software packages. A Structure object holds pointers to Chain instances, while each Chain aggregates Residues. Finally, each Residue is a vector of PdbAtoms. However, unlike other software packages, a Structure instance is not directly created from PDB content; instead, a PDB file reader object is responsible for loading PDB text, parsing it, and creating a Structure from a given model stored in a file. The file reader object provides flexibility that can speed up reading files. The PDB reader takes a PdbLineFilter object as an argument to include or exclude every text line that is loaded from a file. For simplified analysis that requires only mainchain analysis, the core::data::io::is_ca filter can substantially speed-up loading Cα-only coordinates from an all-atom file.

Another typical scenario is loading a very large PDB file that contains a large number of identical models, e.g., resulting from a molecular simulation. These models are frequently processed one-by-one; therefore, creating a Structure object for each of them is an unnecessary burden. Instead, BioShell creates only the first Structure object along with its chains, residues and atoms. Assuming that all the models are chemically identical, all the subsequent structures can be created by solely extracting Cartesian coordinates from PDB text, replacing the respective data fields in the Structure that has been already created. Consequently, all constructor calls and expensive memory allocations are done only for the first model.

### 3.2. Novel Testing Infrastructure

A comprehensive test of sophisticated software is extremely important not only for scientific software. Inadequate testing was blamed for a number of widely publicized accidents [16]. Scientific software is usually more complicated than daily life software, so a novel approach for testing a research software package such as BioShell is a critical part of its development. In the case of a C++ library, such tests are performed by small programs that execute a part of code and compare results with the reference. Although continuous software testing should be a common practice, the testing applications themselves are often hidden from an end user. This is somewhat surprising, given the fact that this code is actually the most exhaustively tested part of the entire package. Here, we propose a novel approach to bring these tests to the stage. More than a hundred test applications have been included in this release to simultaneously reach three goals: (1) to extend the set of BioShell applications, (2) to contribute to unit-testing and the integration testing facility, and (3) to provide examples for scientists who will use the BioShell library in their own applications. All the examples have been organized in three main directories: example_data, cc_examples and py_examples which hold example input files, example C++ applications, and example Python scripts, respectively. Examples are organized to follow C++ namespaces, e.g., tests related to core::calc::structural, such as the ap_Crmsd C++ application can be found in cc_examples/core/calc directory. The set of example input files has been carefully chosen to include well-studied systems important to the field. Relevant files of this set are linked to each test directory, which also contains manually curated results of that test. A list of all these tests (organized by keywords and by their functionality) as well as relevant documentation is automatically updated and hosted on ReadTheDocs website.

### 3.3. Test of Integration and Compatibility between Components

**Figure 2:**
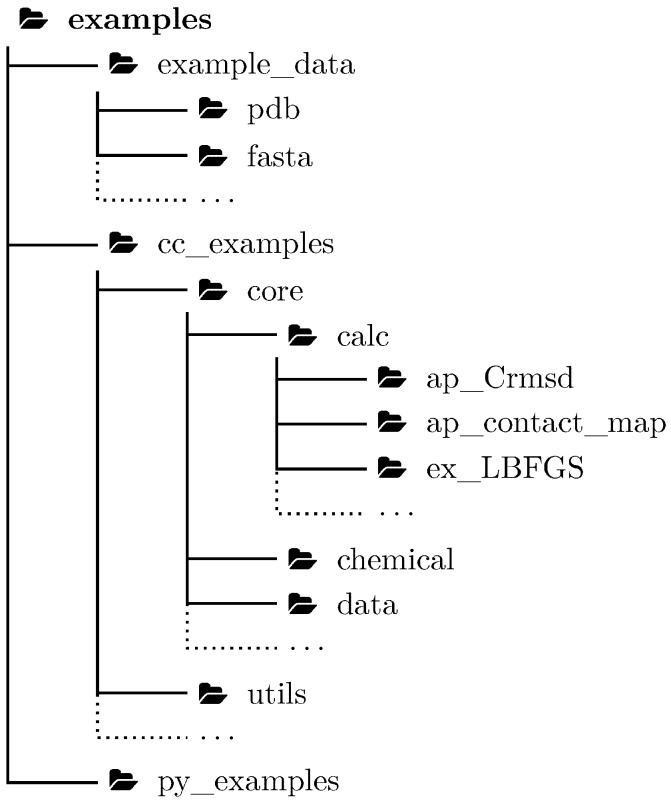
Organisation of BioShell examples.

The standard application development cycle uses integration tests, i.e., small programs written to test compatibility between software’s components. These tests ensure that any changes introduced do not unintentionally negatively affect other parts of the suite. While developing the BioShell package, we attempted to turn as many of these tests as possible into practically useful applications that can be instructive for end users of the package. For each application, biologically relevant input data and the expected output have been provided as part of the repository so these applications may still work as tests (see Figure 2). The name of each program of this group starts with ap_ and is followed by the name of a tested class or module. Occasionally, a program name reflects the functionality it provides rather than the tested class. For instance, ap_stacking_interactions reads a PDB file and prints the relative orientation between any two aromatic rings found in amino acid side chains that are closer than a given cutoff. This small program has been devised to test the core::data::structural::IsAromaticAA residue filter and local reference frame calculations. In another example, the ap_Crmsd application that tests structural superposition can be used to easily calculate crmsd between two PDB files. The simplicity of these apps allows them to be efficiently used in research tasks, such as analysis of MD trajectories, protein structure assessment, or to derive statistical force field.

### 3.4. Unit Tests Serve as Examples for a C++ Library

Unit tests are intended to test a very narrow part of a code, e.g., to invoke a single function. Therefore, in many cases, it was not possible to turn a unit test into a fully functional application. Nevertheless, these tests are also exposed for end users to exemplify the use of BioShell C++ routines; each example’s name consequently starts with ex_. Many programmers create their applications by copying and pasting relevant parts of examples (snippets). In BioShell, the ex_ tests offer a large collection of such short examples showing how to use its most important classes. The code, ready for the copy and paste approach, is regularly compiled and run by a test server, ensuring its correctness.

### 3.5. Comparison with Biopython

Python bindings to the BioShell library introduced by this contribution in some applications can be used to substitute Biopython, especially where computational efficiency is required. Since the latter one is written in an interpreted language (i.e., Python), it will always be slower than a compiled program, and it is difficult to give a direct comparison between the two. Nevertheless, here we provide a few examples to give an impression of what speed up one may expect by replacing Biopython with BioShell in their projects. We choose six problems that can easily be implemented in both Biopython and PyBioShell. They cover the most common tasks in structural bioinformatics, like root mean square deviation calculation for multiple structures or writing just the C-alpha atoms from a full-atom PDB file. The role of each test script is as follows:

- ca_only_multimodel—reads multiple PDB files with a single model and writes them into one file using only the C-alpha atoms’ coordinates;
- contact_map—checks which residues are within a given distance to each other and returns a list of neighbors with the number of contacts found in a multi-model PDB file;
- pdb_to_fasta—prepares biopolymer sequences in fasta format from a PDB file;
- ramachandran—returns information about phi and psi angles and amino acid type (Glycine, Pre-Proline, Proline or General);
- read_pdb—reads a pdb file;
- rmsd—calculates root mean square deviation between C-alpha atoms of two PDB files.

**Figure 3:**
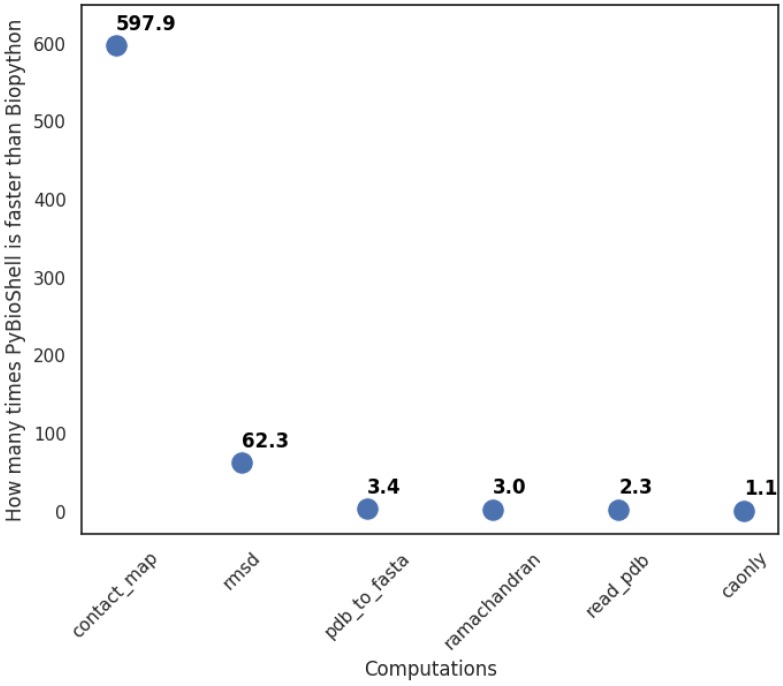
Results of PyBioShell and Biopython comparison.

We repeated each test twenty times with an internal time measurement. In two cases, PyBioShell was significantly faster than Biopython (see Figure 3). The reason is the improved PDB file reading and storage mechanism implemented in BioShell 3.0.

## 4. Discussion

Throughout its history, the BioShell package has been applied to numerous problems studied by several groups all over the world. In this contribution, we described the newest, greatly improved and expanded version of the software. The library extends the Python scripting language with a robust and powerful interface to investigate and manipulate protein structures, sequences and alignments. With the widespread use of Python in bioinformatics, the package will certainly find new applications, especially among users who are not programming experts. Extensive documentation, comprising a detailed description of the package, a cookbook of most popular commands, a rich library of examples, API documentation, and tutorials certainly make it easy to approach. When compared to Biopython, it offers a significant improvement in execution time. BioShell therefore can be used in high throughput computations as well as for interactive work, e.g., as a component of web servers for bioinformatics applications.
